# Making sense of discrepancies in working memory training experiments: a Monte Carlo simulation

**DOI:** 10.3389/fnsys.2014.00161

**Published:** 2014-09-02

**Authors:** David Moreau

**Affiliations:** Department of Psychology, Princeton UniversityPrinceton, NJ, USA

**Keywords:** cognitive training, working memory, individual differences, intelligence, Monte Carlo simulation, random sampling

## Introduction

The idea that working memory training can enhance general cognitive abilities has received a lot of attention in the last few years. Some studies have demonstrated far transfer to other cognitive abilities after training, whereas others have failed to replicate these findings (see Melby-Lervåg and Hulme, 2013, for a meta-analytic review). These setbacks have had unfortunate effects on the field of cognitive training, sometimes raising concerns regarding methodology and analyses in concurrent labs. Although constructive skepticism is often healthy in fields based on peer-review, the current dynamic has created a climate of hostility detrimental to the advancement of science. In this paper, I show that some of these differences can simply emerge from population parameters underlying cognitive growth curves. Based on a Monte Carlo simulation, I demonstrate that unbalanced samples are bound to arise by chance when individuals differ in their ability to learn, and propose a few remedies to circumvent this problem. Finally, I discuss the impact of cognitive training studies in refining theories of cognition and suggest directions for future research.

## Working memory training and individual differences

Working memory capacity (WMC) is at the core of numerous mental operations, including reasoning, problem solving and decision-making. In line with this idea, recent advances using computational modeling have shown that WMC correlates highly with a wide range of cognitive constructs, including *g* (Süß et al., 2002; Kane et al., 2004), an idea further supported by common neural correlates for WMC and *g*, particularly regions of the prefrontal cortex (Kane and Engle, 2002; Gray et al., 2003). Although a strong correlation between constructs does not guarantee that they will covary with training (Moreau and Conway, 2014), the relationship between WMC and *g* was the starting point for a vast enterprise aimed at increasing the fluid component of intelligence (Gf) via working memory training (Jaeggi et al., 2008). However, since early studies showing improvements in tasks tapping Gf after working memory training (Jaeggi et al., 2008, 2010; Jaušovec and Jaušovec, 2012), others have consistently failed to replicate these findings (Chooi and Thompson, 2012; Harrison et al., 2013; Redick et al., 2013). These contradicting results created a dichotomy between labs interested in the same trend of research but reaching different conclusions. As of today, many would say that the jury is still out concerning the effectiveness of working memory training to improve Gf, and that previous shortcomings need to be addressed.

One factor of particular importance in this debate concerns individual differences in learning curves. This point has been emphasized recently, with researchers suggesting that understanding differential effects is critical to better assess and design cognitive training programs (e.g., Jaeggi et al., 2014). This is a healthy departure from dichotomized claims about the effectiveness of working memory training, illustrating the importance of more nuanced statements—the same training does not work for everyone, and it is critical to determine what components are required for successful transfer and what components need to be adapted to individual needs. Until we can successfully identify these parameters, training programs will yield differential effects that are difficult to predict.

Yet how and to what extent do individual differences influence training outcomes? In the following section, I argue that individual differences in rates of improvement can induce differences in the measured outcomes simply due to random sampling from heterogeneous populations—assuming such a scenario, a non-trivial number of experiments will already be biased at the onset. Regardless of the presence of a true effect of training, sampling errors can obscure comparisons between conditions because of unbalanced samples, which in turns can create the illusion of an effect or of the absence of an effect when in fact the opposite is true.

## A monte carlo approach

A Monte Carlo simulation helps to understand this idea. A typical training experiment aims for an *N* of about 20 subjects per cell, so we will consider the case of training experiments with two groups, experimental and control, with a total of 40 subjects. Experimenters usually sample the population until the desired number of subjects is reached, at which point they are randomly assigned to either an experimental or a control group. Recruiting all subjects before random assignment allows starting training at the same time for all, often to provide more control over external factors, and ensuring that group samples are equivalent on the tasks of interest measured at pre-test. An alternate solution is to assign subjects to a group at the recruiting stage, in order to start training at each subject's earliest convenience. This is often more practical when experimenters have to deal with time constraints, but it can potentially introduce additional confounds. Because the present simulation does not constrain sampling hierarchically, its results will not be influenced by the sampling strategy one might favor.

A simulation with 10,000 draws was performed for each of these scenarios: uniform population, population with two subpopulations, and population with three subpopulations. In the first step of the simulation, all subpopulations were equally represented in the overall population—the only variation allowed in the model was at the random sampling stage.

Assuming the overall population we are sampling from is uniform, dividing a sample into two groups (experimental vs. control) has no effect on how balanced a design is. This is what was is mostly assumed in training studies that are not focused on individual differences, either explicitly or implicitly, because of the nature of the design itself. When assuming such an underlying population, random assignment to experimental conditions never produces unbalanced samples—the population is uniform, and so are the two samples.

**Figure 1:**
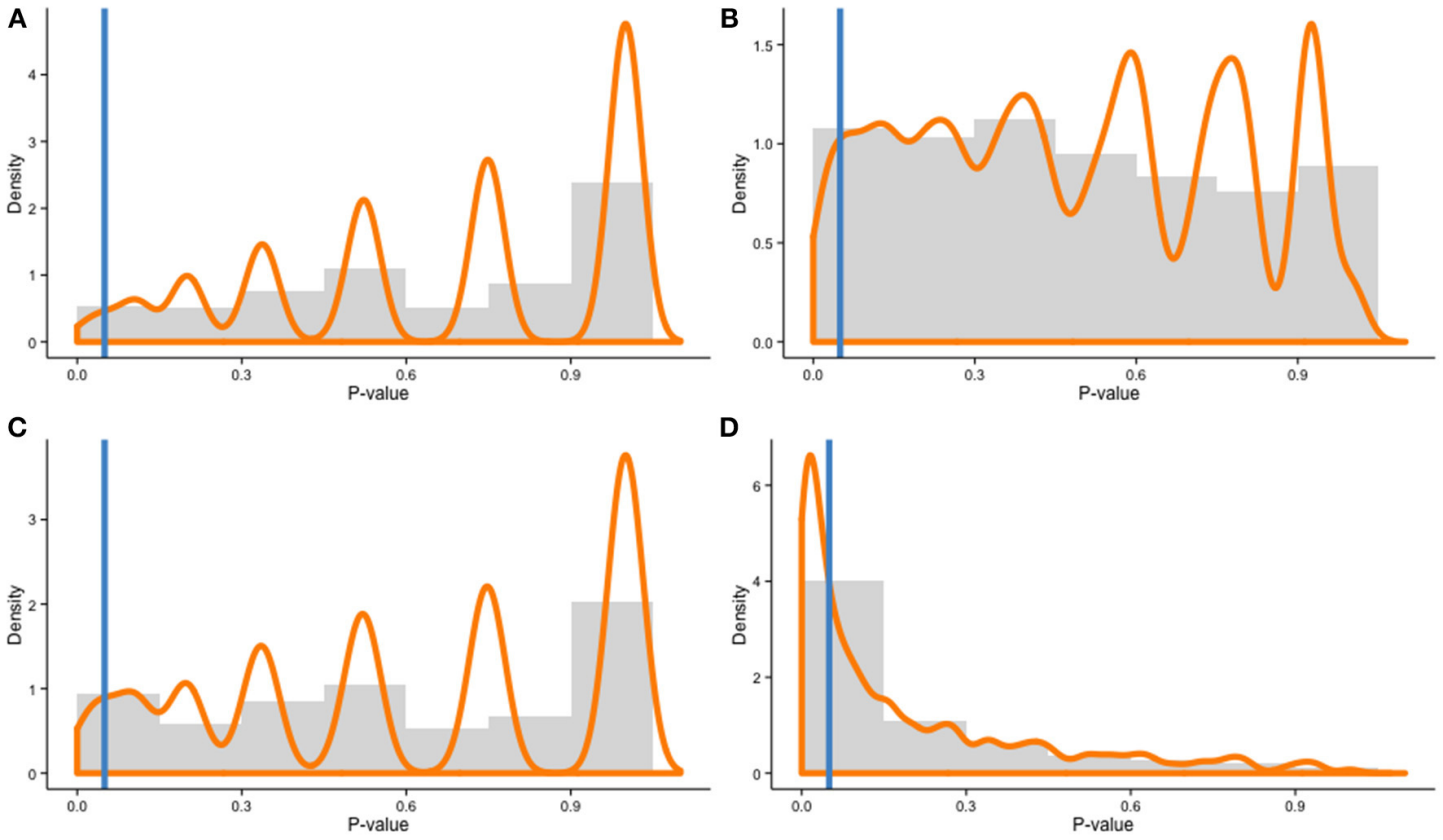
**Distributions of χ-squared contingency table test *p*-values for a Monte Carlo simulation with 10,000 draws of two samples (*N* = 20 per cell) in four different scenarios: (A) 2 subpopulations—Equal ratios, (B) 3 subpopulations—Equal ratios, (C) 2 subpopulations—Unequal ratios (60–40%), and (D) 3 subpopulations—Unequal ratios (55–35–10%)**. Histograms represent distribution frequencies; the orange line depicts density estimates. The blue line represents the threshold for *p* = 0.05 (all *p*-values to the left of the line are significant, indicating unbalanced samples).

Recent evidence, however, suggests different learning curves, or rates of improvement, between individuals, based on distinct neural changes (e.g., Kundu et al., 2013). This is a completely different scenario. Let us assume the general population we are sampling from includes two subpopulations with different rates of improvement (high and low). In this case, a Monte Carlo simulation with 10,000 draws shows that random sampling from the population will yield unbalanced samples 1.74% of the time (Figure 1A). This percentage might seem trivial, but when the population includes three subpopulations of learners (high, medium, low), a simulation with 10,000 draws yield unbalanced samples 5.18% of the time (Figure 1B). The probability rises quickly when more subpopulations are included in the model: the more individuals differ in their ability to learn, the more likely a training experiment is to be affected by sampling error. In fact, ecological populations are likely to be even more heterogeneous, therefore exacerbating this effect. In training experiments, individual differences matter.

Such sampling errors are substantial, especially given that the model proposed here did not assess the probability for a sample to represent adequately the overall population, but only the probability for two samples drawn from the same population to be equivalent. Moreover, sampling errors and other limitations common with the analyses of training data (e.g., correlated gains and dichotomization; Tidwell et al., 2014) are not mutually exclusive, arguably increasing the risk for experimental confounds. The underlying rationale in training experiments is that sampling error is random, and that it therefore averages with large enough samples. However, despite inferential tools available to estimate the size of sampling error (e.g., standard error), unbalanced samples often go undetected, because no effort is made to test for homogeneity and the distribution of the population is rarely known *a priori*.

One should also note that the percentages presented here are conservative—subpopulations within the overall population were always equally represented in the model, but this is not necessarily the case in real settings. In fact, it is plausible that individuals with different trends of improvement are not equally represented in the overall population. In this case, the probability to draw unbalanced samples rises quickly. In the two-subpopulation scenario, unbalanced overall population with the following ratios (high = 60%; low = 40%) yields unbalanced samples across groups 4.23% of the time with 10,000 draws (Figure 1C). In the three-population scenario with the following ratios (high = 55%, medium = 35% and low = 10%), the probability surges to 37.87% (Figure 1D). Given an unequal percentage of each subpopulation within the overall population, it can be more accurate to use a stratified sampling method to improve representativeness of the samples; however, this requires knowing *a priori* the percentage of each subpopulation in the overall population to be accurate, which is often difficult in practice.

## Potential remedies

There are a few remedies to circumvent this problem. First, the simulation presented in this paper demonstrates the importance of assessing learning rates before training starts. In this regard, specific tasks could be used to define a growth rate in the ability of interest (e.g., WMC), allowing experimenters to introduce specific constraints in a pseudo-random assignment to groups, such as forcing matched samples. This additional step would ensure balanced samples across experimental conditions at the onset of the study to reduce the potential biases emphasized in this paper. In addition, and because they would be measured before any experimental treatment, initial growth profiles could be used as covariates in the final analyses to refine the interpretation of significant effects.

Testing for differences in learning rates also emphasizes the importance of sample size. All other parameters being equal, a larger sample size increases the probability to detect differences in growth profiles between experimental groups (i.e., power). For example, increasing the sample in the Monte Carlo simulation to 40 subjects per cell allows detecting unbalanced samples in 3.47% of cases in the two-subpopulation scenario (11.05% with unequal subpopulations), and in 5.20% of cases in the three-subpopulation scenario (69.47% with unequal subpopulations). Obviously, the cost of training experiments combined with the desire to publish findings quickly represent strong incentives for experiments with smaller sample size, but the field of cognitive training needs more statistically powered designs to reach more definitive claims.

Finally, predefining a one-sided hypothesis at the onset of a study allows reducing the probabilities defined in previous scenarios by half, since half the time the discrepancies arising spuriously will contradict a specific hypothesis. Preregistering is now made easier by online projects such as the Open Science Framework (http://openscienceframework.org), which keeps time stamps on project submissions and allows choosing an appropriate level of privacy for a preregistered project. Furthermore, top-tier journals in psychology and neuroscience are now encouraging such preregistrations, by approving particular designs and hypotheses before an experiment is conducted, therefore guaranteeing publication regardless of the outcome. Clearly, these initiatives reach far beyond solving the problem of ambiguous hypotheses—for example, they also represent a first step toward eradicating the file-drawer problem—and in that regard they should be widely applauded and further encouraged.

## Concluding remarks

The field of cognitive training is an exciting one, with tremendous potential applications. In this regard, inconsistencies in experimental findings should not be interpreted as failures to advance knowledge but as inevitable consequences of exploring a field still in its infancy. Importantly, I do not argue that sampling error explains *all* discrepancies in working memory training outcomes; rather, the rationale for this opinion piece is to encourage caution when interpreting seemingly incompatible findings. Moreover, the limitation presented herein, as well as its potential remedies, are equally valid to other types of training designs not based on working memory—in fact, I do hope that the paper contributes to an already ongoing shift of focus from general training contents to more individualized programs, taking into account individual differences in cognition. Following this idea serves a dual purpose—it allows designing more effective training programs with applications to clinical and non-clinical populations, particularly important in our aging societies, but also provides suitable environments to test empirical claims and refine current models of cognition.

### Conflict of interest statement

The author declares that the research was conducted in the absence of any commercial or financial relationships that could be construed as a potential conflict of interest.
